# DNAJB6 Myopathies: Focused Review on an Emerging and Expanding Group of Myopathies

**DOI:** 10.3389/fmolb.2016.00063

**Published:** 2016-09-30

**Authors:** Alessandra Ruggieri, Simona Saredi, Simona Zanotti, Maria Barbara Pasanisi, Lorenzo Maggi, Marina Mora

**Affiliations:** Neuromuscular Diseases and Neuroimmunology Unit, Fondazione IRCCS Istituto Neurologico Carlo BestaMilan, Italy

**Keywords:** DNAJB6, chaperone, LGMD1D, distal myopathy, autophagy, protein aggregation, vacuolar myopathy

## Abstract

Mutations in the *DNAJB6* gene have been associated with the autosomal dominant limb girdle muscular dystrophy type 1D (LGMD1D), a disorder characterized by abnormal protein aggregates and rimmed vacuoles in muscle fibers. DNAJB6 is a ubiquitously expressed Hsp40 co-chaperone characterized by a J domain that specifies Hsp70 functions in the cellular environment. DNAJB6 is also a potent inhibitor of expanded polyglutamine (polyQ) aggregation preventing aggregate toxicity in cells. In *DNAJB6*-mutated patients this anti-aggregation property is significantly reduced, albeit not completely lost. To elucidate the pathogenetic mechanisms underlying the DNAJB6-related myopathy, animal models have been created showing that, indeed, conditional muscular expression of a DNAJB6 mutant in the mouse causes a LGMD1D myofibrillary muscle tissue phenotype. Both mutations and phenotypes reported until recently were rather homogeneous, being exclusively missense mutations of a few amino acids of the protein G/F domain, and with a phenotype characterized by adult-onset slowly progressive muscular dystrophy predominantly affecting proximal muscles. Lately, several novel mutations and new phenotypes of DNAJB6 have been described. These mutations once more affect the G/F domain of DNAJB6 with missense changes and a splice site mutation; and the phenotypes include childhood onset and distal involvement of muscles, or childhood-onset LGMD1D with loss of ambulation in early adulthood and respiratory involvement. Thus, the spectrum of *DNAJB6*-related phenotypes is widening. Although our knowledge about the role of DNAJB6 in the pathogenesis of muscle diseases has made great progression, several questions remain unsolved, including why a ubiquitous protein affects only, or predominantly, skeletal muscle; why only the G/F domain is involved; and what is the possible role of the DNAJB6a isoform. Clarification of these issues will provide clues to implement possible therapeutic strategies for DNAJB6-related myopathies.

## Introduction

The first description of what it was, at that time, defined as “an unusual form of muscular dystrophy” dates back to 1969, when Schneiderman et al. (1969) described a four generation family affected by a late onset dominant muscular dystrophy form, predominantly affecting proximal limb muscles. Neither the genetic cause of this dystrophy nor the locus were at that point clarified.

A step forward on the identification of the causative gene for this form of limb girdle muscular dystrophy (LGMD), now named LGMD1D, was taken by Speer et al. (1999) who provided evidence of linkage to the 7q locus. In this study Speer and colleagues gathered five families based on the presence, in the affected members, of progressive proximal lower limb muscle weakness with or without proximal upper limb involvement, increased creatine kinase (CK) levels, absent ankle deep tendon reflexes, and no features suggestive of any other known myopathy, by muscle histology and electron microscopy. Genome wide linkage excluded three of the five families establishing evidence for linkage to 7q for the remaining two, previously described by Schneiderman et al. (1969) and Speer et al. (1995). Two recombination events in both families allowed the authors to better define the region in an interval of 9 cM comprised between markers D7S2546 and D7S2423.

Few years passed by before new LGMD1D families were reported in 2010 (Sandell et al., 2010). One year later, Hackman et al. (2011) collected four informative new Finnish families leading the way to the identification of the genetic cause of LGMD1D. The presence of informative recombination in two unaffected members of two of these families allowed reducing the 7q region to 3.4 Mb, containing 12 known genes, and at least 14 hypothetical genes or pseudogenes. Subsequently, a candidate gene sequencing approach let Sarparanta et al. (2012) to identify the LGMD1D causative gene.

With the discovery of novel mutations in the *DNAJB6* gene, the spectrum of related phenotypes, in terms of age of onset, severity and group of muscles involved, is widening.

In the present review we will focus our attention on the pathological effects of mutations affecting the DNAJB6 chaperone protein, and on the clinical and histopathological features of the DNAJB6-related myopathies.

## Molecular aspects of DNAJB6

Cells, at each stage of their life, depend on the essential support of proteins as building blocks and to carry out all cellular functions. Proteins have a proper three-dimensional conformation, which, as demonstrated by *in vitro* experiments (Anfinsen, 1973), depends on the amino acid sequence and can be achieved spontaneously according to the global minimum of free energy. However, the experimental conditions required for a proper *in vitro* folding are very restrictive and not applicable to the crowded cellular environment where hydrophobic effects will make harder to control the folding. Moreover, this process is challenged by various stress conditions, some, such as the increase in protein synthesis during cell cycle progression, constitutive; others, such as environmental or pathophysiological stresses (e.g., temperature increase or tissue injury and repair), sporadic. Therefore, in order to prevent the formation of toxic protein aggregates, the cell requires an active and dynamic system able to control proper protein folding and clearance of the misfolded and damaged proteins.

Molecular chaperones are part of this dynamic system that helps maintaining cellular protein homeostasis through their ability to interact among themselves and with specific partners, thus influencing conformation and function of a wide range of different substrates such as p53 and other transcription factors, including steroid receptors, as well as proteins that unfold and aggregate in neurodegenerative diseases (polylglutamine androgen receptor, huntingtin, α-synuclein, tau) and a variety of protein kinases (Morimoto, 2008; Pratt et al., 2015). They are named heat shock proteins (HSPs) and grouped into families according to their molecular weight: Hsp100, Hsp90, Hsp70, Hsp60, Hsp40, and sHsp (small heat-shock protein). The Hsp70 chaperones are involved in a plethora of processes including folding of newly synthesized proteins, transport of proteins across membranes, refolding of misfolded and aggregated proteins, and control of regulatory protein activity (Bukau et al., 2006). Hsp70 chaperones have a 40 kD N-terminal ATPase domain and a 25 kDa C-terminal peptide-binding domain (PBD), and cycle between ATP- and ADP-bound conformation. In the ATP form, the bond between client polypeptides (newly synthesized or misfolded proteins) and the PBD of Hsp70, is weak.

**Figure 1:**
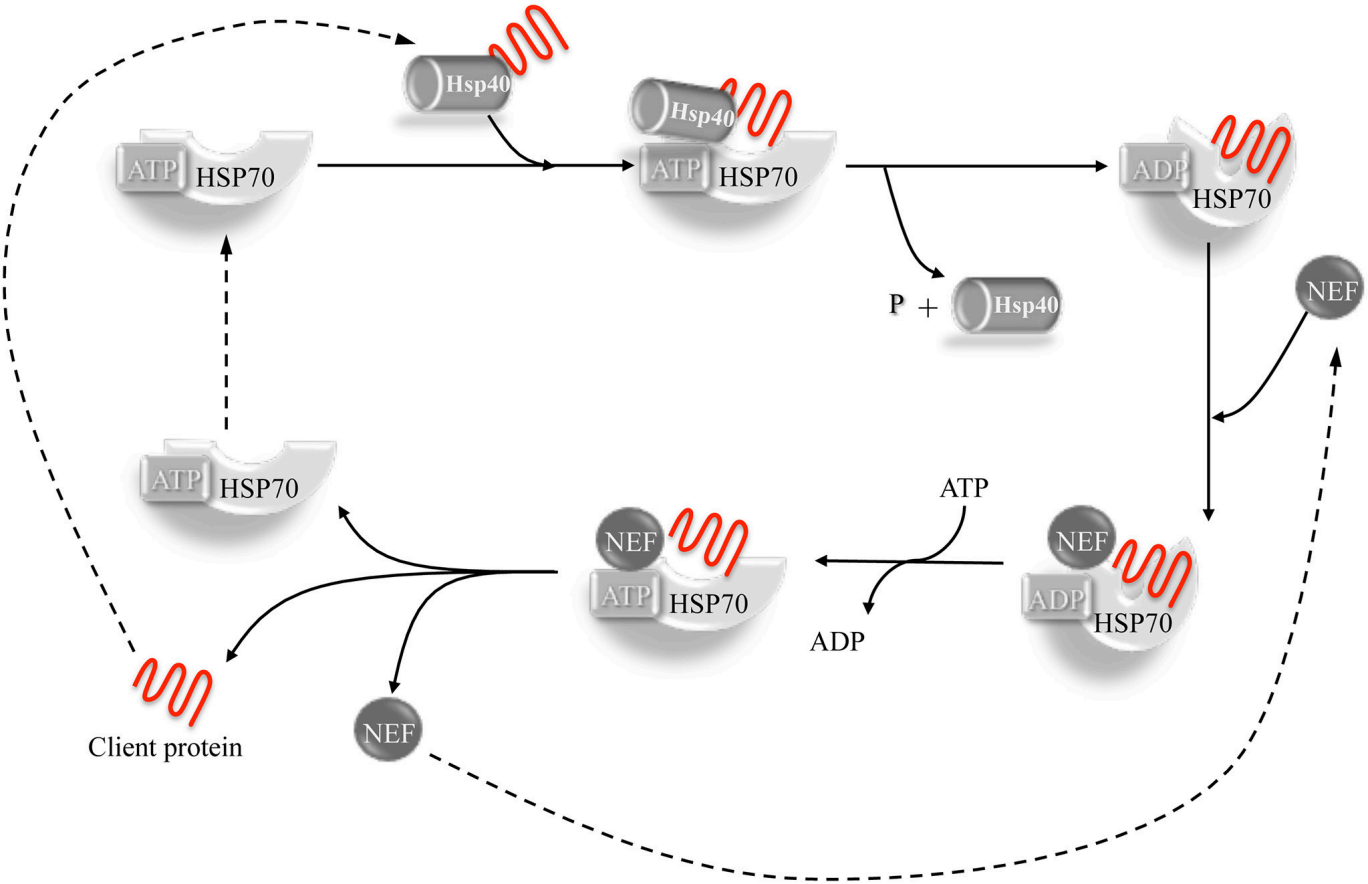
**The Hsp40-Hsp70 cycle**. Hsp40 co-chaperone forms complexes with unfolded or non- native proteins delivering them to Hsp70. The interaction between Hsp40 and the ATP-bound Hsp70 takes place through the J-domain. The client protein transiently interacts with the Hsp70 and the interaction is stabilized by a conformational change in Hsp70 caused by the hydrolysis of ATP which, in turn, is stimulated by both Hsp40 and client protein. Hsp40 is then released. A nucleotide exchange factor (NEF) then binds Hsp70 (having more affinity for the ADP-bound form compared to the ATP form), stimulating the dissociation of ADP through a conformational change in Hsp70. An ATP molecule is bound again to Hsp70 because of its higher cellular concentration, causing the release of the client protein. The system is then ready for a new cycle (Kampinga and Craig, 2010).

The chaperone-client polypeptide interaction is stabilized by the intervention of co-chaperone proteins belonging to the DnaJ family (Hsp40). The DNAJ co-chaperones associate with the client proteins presenting them to the Hsp70 chaperone, thus leading to the formation of a trimeric complex. Co-chaperone plus substrate stimulate the Hsp70 dependent hydrolysis of ATP to ADP with consequent conformational change of the Hsp70 protein that increases its affinity for the substrate and triggers the separation of the DnaJ co-chaperone. The release of the client protein is then achieved by the dissociation of ADP, stimulated by nucleotide exchange factors (NEFs), allowing the Hsp70 chaperone to be ready for a new cycle (Laufen et al., 1999; Kampinga and Craig, 2010; Figure 1).

**Figure 2:**
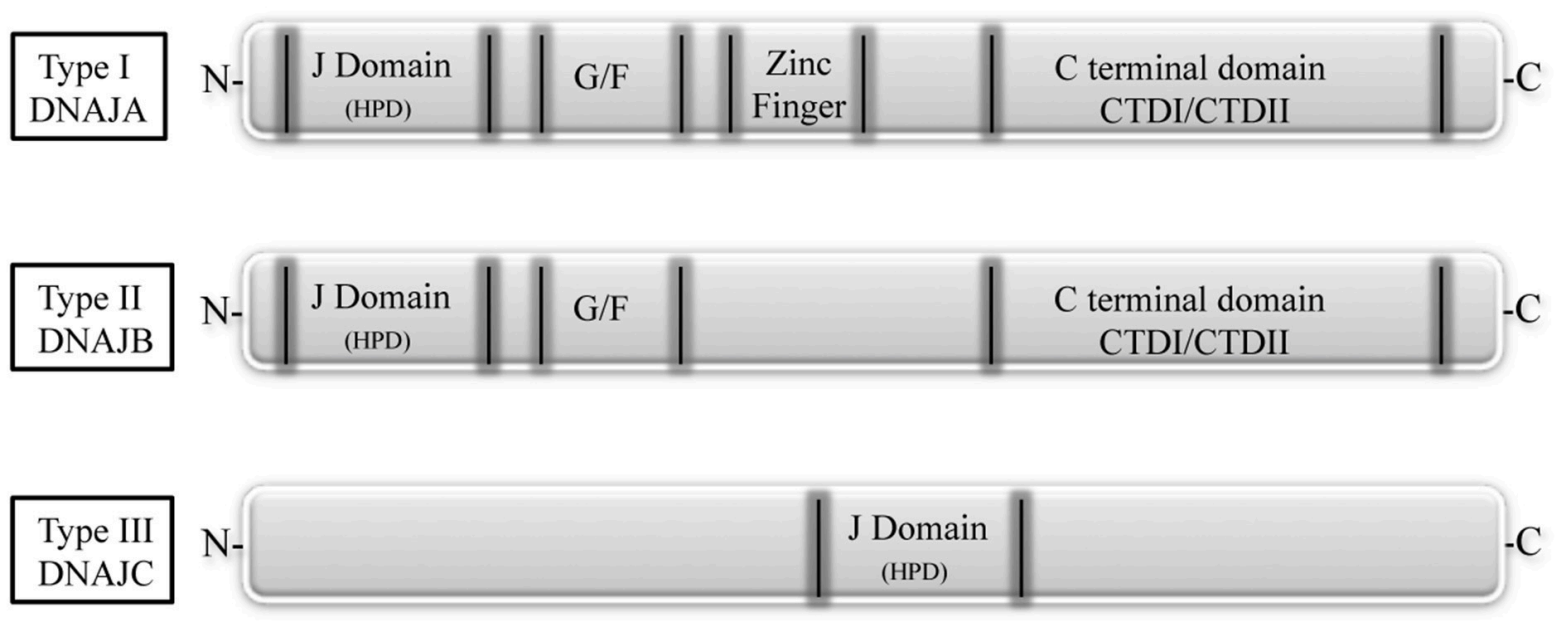
**The DNAJ protein family**. Based on their structure, DNAJs are divided into three subtypes. Type I, or A, has an N-terminal J domain (with a conserved histidine, proline, and aspartic acid tripeptide), followed by a glycine/phenylalanine-rich domain (G/F), by a zinc finger region rich in cysteine, and by a C-terminal domain, responsible for client binding and presenting two barrel domains, CTDI and CDTII. Type II, or B, is similar to type I, but lacks the zinc finger region. Type III, or C, has only the conserved J domain which is variably located in the protein (Fan et al., 2003).

DNAJ/Hsp40 co-chaperones are a diverse and large group of proteins characterized by the presence of a 70 amino acid sequence, the J domain, as common signature. The J domain stimulates the Hsp70 ATPase activity and contains a conserved tripeptide sequence (histidine, proline and aspartic acid, HPD) critical for its function. The Hsp40 family is divided into three subtypes according to their structure (Figure 2). The type I, or A, is closely related to the *E. coli* DnaJ and comprises the J domain at the N-terminus, a glycine/phenylalanine (G/F)-rich domain, a cysteine-rich region, and a C-terminal region that recognizes and binds to the substrate. The direct function of the G/F domain is not clear. A likely one is that the G/F domain participates in the recognition and modulation of particular substrates, thus acting on the specification of Hsp70 function (Fan et al., 2003). The DNAJ type II or B, has similar structure to type A, but lacks the cysteine-rich domain. The type III, or C, contains, as conserved region, only the J domain, which can be localized anywhere in the protein (Li et al., 2009; Kampinga and Craig, 2010).

Even though it has been demonstrated that the J domain alone is capable of stimulating the ATPase activity of Hsp70, the presence of a functional Hsp70 PBD is required to allow the formation of a complex between the unfolded protein and Hsp70 (Fan et al., 2003). DNAJ proteins, promote multiple functions of Hsp70 by binding and delivering a variety of non-native clients. Some DNAJ chaperones are involved in the correct folding of the newly synthesized proteins (DNAJA1 and DNAJB1), some have a role in delivering misfolded proteins to the nucleus for proteasomal degradation (DNAJB1), some (DNAJB9, DNAJC10, DNAJB11) intervene in the endoplasmic-reticulum-associated protein degradation (ERAD) pathway, recognizing misfolded proteins that are consequently ubiquitinylated and degraded (Park et al., 2013; Dekker et al., 2015).

A distinctive characteristic of DNAJB6 and DNAJB8 is their capability to act as powerful inhibitors of misfolded poly-Q protein aggregation, which is dependent on and occurs through direct interaction with the C-terminus SSF-SST serine-rich region (Hageman et al., 2010; Gillis et al., 2013). The purified DNAJB6 protein is able to inhibit the formation of pathogenic polyQ aggregates at a substoichiometric molar ratio and independently from the presence of Hsp70 and ATP (Månsson et al., 2014). Unlike in purified protein, in living systems there is need for interaction between DNAJB6 and Hsp70 in those situations in which the DNAJB6 anti-aggregation capability is somehow limited (Kakkar et al., 2016).

This thorough study by Kampinga's group also demonstrated that the most efficient anti-aggregation activity is dependent on an array of 18 hydroxyl groups in the S/T-rich region of DNAJB6. Additional evidence of DNAJB6 anti-aggregation property comes from demonstration that overexpression of the human DNAJB6b in yeast could directly prevent [URE3] prion formation and block the propagation and spontaneous formation of Ure2 amyloid fibers (Reidy et al., 2016). This further proof indicating DNAJB6 as anti-aggregant acting on structurally different types of amyloids, makes DNAJB6 an attractive therapeutic target in amyloid storage disorders.

In general, because of their involvement in the diverse tasks of protein homeostasis control, DNAJ proteins are also clinically relevant disease targets. In fact, mutations leading to disease have been found in seven distinct DNAJ proteins, comprising DNAJB2, DNAJB6, DNAJC5, DNAJC6, DNAJC13, DNAJC19, and DNAJC29 (Koutras and Braun, 2014).

**Figure 3:**
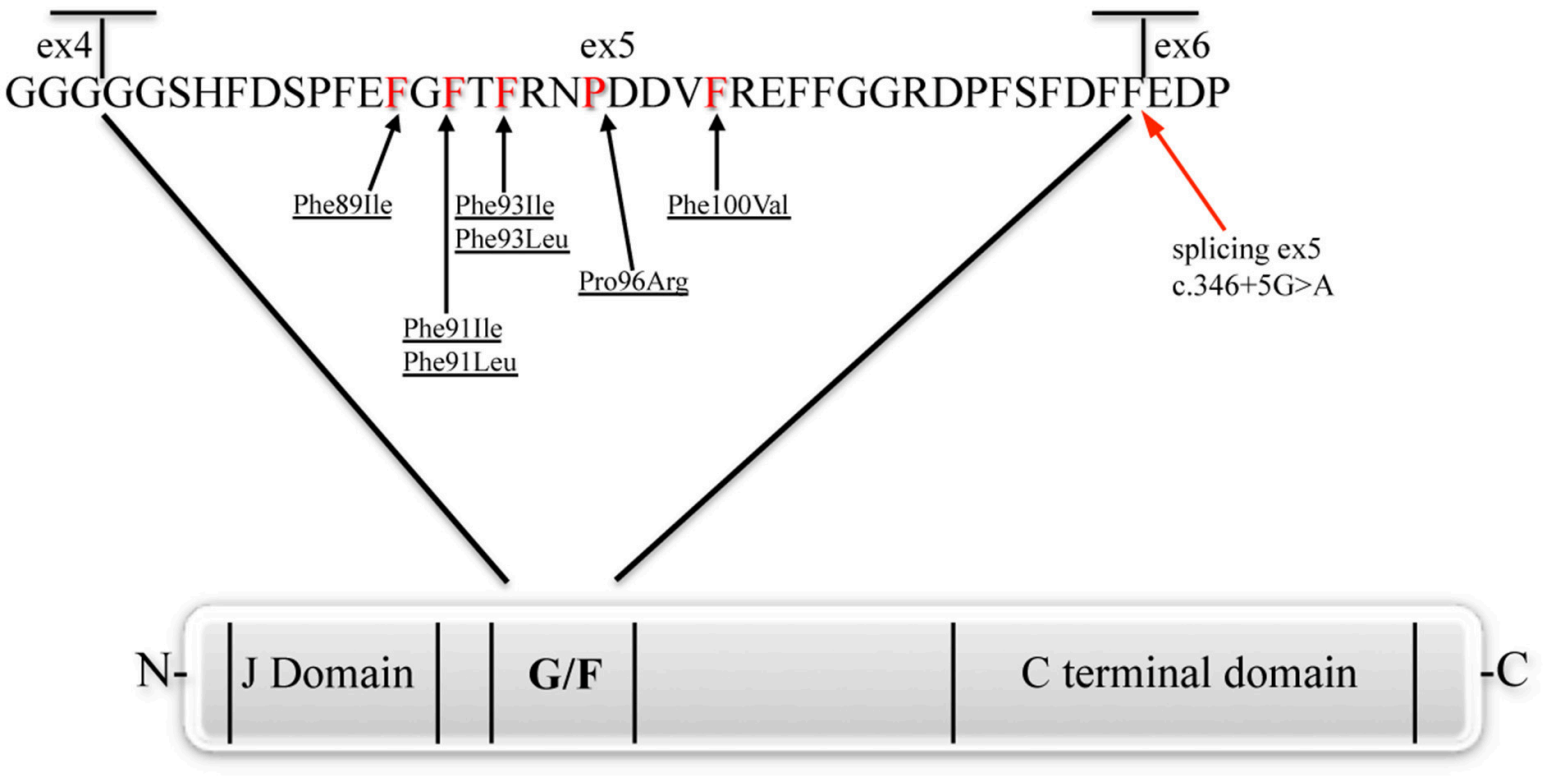
**The DNAJB6 protein**. The J, G/F and C-terminal domains, and position of the amino acid and splicing mutations are represented (Fan et al., 2003; Sato et al., 2013; Ruggieri et al., 2015).

The present review will focus on mutations affecting the chaperone DNAJB6 (Figure 3), causing limb-girdle muscular dystrophy type 1D (LGMD1D) and, as shown by recent reports, other forms of myopathy affecting distal muscles. DNAJB6 is ubiquitously expressed as two isoforms with distinct cellular localizations: The longer DNAJB6a localizes to the nucleus and the shorter DNAJB6b localizes to both the cytoplasm and the nucleus.

In the thorough work of Udd's group (Sarparanta et al., 2012), the molecular mechanisms explaining the dominant form of LGMD1D affecting Finnish, American, and Italian patients sharing the common 7q36 locus, were investigated. These authors showed that, in muscle tissue, DNAJB6 protein is primarily localized at the Z-disks, and that in LGMD1D patients a Z-disk myofibrillar disintegration was visible with accumulation of DNAJB6 and its known ligands MLF1 (myeloid leukemia factor 1), HSPA8 and KRT18 (keratin 18). With the use of morpholino (MO) in *in vivo* experiments on zebrafish, they were able to demonstrate loss of muscle integrity caused by the silencing of *dnajb6b* (the unique ortholog of the human gene), impairment visible already at 2 days post fertilization in injected embryos. Expression of the human wild-type DNAJB6 could rescue the phenotype thus demonstrating that loss of DNAJB6 was the direct cause of muscle defects. Furthermore, to investigate the effects of specific mutations on the a and b isoforms, human mutated transcripts generated for both isoforms were injected into zebrafish embryos showing that only the b isoform recapitulates the muscular phenotype observed with the dnajb6 splice blocking morpholino. Moreover, Sarparanta and coll. showed that the molar ratio of mutant to wild-type *DNAJB6* mRNA is crucial: An equimolar injection of mutant and wild type *DNAJB6* messenger will increase the severity of the phenotype, suggesting a dominant effect. An excess of mutant over wild-type is lethal for embryos, while the opposite allows a progressively increasing rescue. Using a filter trap assay, the mutant proteins were shown to have reduced anti-aggregation properties of polyglutamine-containing huntingtin (pEGFP/HD-120Q), compared to wild-type DNAJB6 protein, confirming data previously found by Hageman et al. (2010). The reported mutations, although not affecting the S/T-rich region proven to be responsible for the anti-aggregation activity (Kakkar et al., 2016), might indirectly alter this property—e.g., modifying the protein conformation and impairing the interaction with different clients—thus causing protein accumulation in patient's muscles and variability of clinical severity.

Because of the Z-disk localization and the interaction of DNAJB6 with HSPA8, one of the components of the chaperone-assisted selective autophagy (CASA) complex (Izawa et al., 2000; Arndt et al., 2010), the authors investigated the possible link between DNAJB6 and CASA. By co-immunoprecipitation and proximity ligation assay, the authors showed interaction of DNAJB6 with the components of the CASA complex BAG3 (a protein found to be mutated in myofibrillar myopathy type 6), HSPB8 and STUB1, but none of the interactions appeared to be affected by the p.Phe93Leu DNAJB6 mutation. The authors concluded that the myofibrillary muscle tissue phenotype of LGMD1D patients could be due to an inefficient maintenance of the sarcomeric structure or an increased accumulation of misfolded sarcomeric proteins caused by the impairment of the CASA system, although experimental evidence of a direct role of DNAJB6 in the CASA machinery has yet to be provided.

Another important work investigating the effects of the LGMD1D mutations, by Stein et al. (2014), exploited the yeast ortholog Hsp40 Sis1, creating a Sis1-DNAJB6 protein chimera in which the G/F domain of DNAJB6 was substituted to that of Sis1. With the use of the prion strains of [*RNQ*+] and [*PSI*+], Stein and coworkers, showed that, compared to the wild type chimera, in the LGMD1D mutant chimera the ability to process the protein aggregate conformers was impaired. They also investigated these findings in a mammalian system by monitoring TDP-43 aggregate formation in HeLa cells upon heat-shock and during recovery. TDP-43 contains a C-terminal prion-like domain (Fuentealba et al., 2010) and forms aggregates in skeletal muscle of *DNAJB6*-mutated patients (Harms et al., 2012). In Hela cells expressing TDP-43 and DNAJB6 mutations and in primary patients' fibroblasts, TDP-43 aggregates persisted after heat-shock and recovery, but not in Hela cells expressing DNAJB6-wild type and in control fibroblasts, thus confirming previous suggestion that TDP-43 is a client protein of DNAJB6 (Udan-Johns et al., 2014). These authors proposed that a different degree of selectivity for substrate conformers would be conferred to the co-chaperone protein by the G/F domain: Different mutations in this domain would disrupt the substrate selectivity differentially, thus providing an explanation for the diverse clinical presentations of *DNAJB6*-related myopathies.

## Animal models

In addition to the zebrafish utilized by the Udd's group (Sarparanta et al., 2012) to demonstrate that *DNAJB6* was the gene responsible for LGMD1D, very recently the *Drosophila melanogaster* has been used to establish a mechanistic link between the human genes *DNAJB6* and *hnRNPA2B1* (causing multi system proteinopathy and autosomal dominant familial amyotrophic lateral sclerosis) (Li et al., 2016). These authors generated transgenic *Drosophila* lines either carrying mutations in *Hrb98DE* (the fly homolog of *hnRNPA2B1*) or in *MRJ* (the fly homolog of human *DNAJB6*), in amino acids corresponding to those mutated in LGDM1D patients. They demonstrated that the cytoplasmic aggregation and mislocalization of the mutant Hrb98DE and hnRNPA2B1 proteins, was rescued by co-expressing the wild-type *MRJ* but not the disease-associated *MRJ* mutant. In addition, partial reduction of the endogenous *MRJ* levels, obtained using a classical loss of function line, caused great increase in cytoplasmic aggregation of the mutant Hrb98DE. Finally, the authors showed that an intact G/F domain is indispensable for controlling the formation of mutation-dependent Hrb98DE or hnRNPA2 cytoplasmic aggregates.

The first study of *DNAJB6* homolog gene in mouse was published in 1999 (Hunter et al., 1999). These authors identified, by gene trap screen, the *Mrj* (mammalian relative of DnaJ) gene and characterized its expression during mouse development, showing that *Mrj* is expressed throughout the trophoblast lineage with higher levels in the trophoblast giant cells of the placenta. The homozygous *Mrj* mutants were lethal because of a failure in chorioallantoic fusion at embryonic day 8.5. Eight years later Watson et al. (2007) demonstrated that absence of a functional Mrj protein in trophoblast cells, causes the deposition of keratin aggregates that disrupt cell function and organization, leading to defects in chorioallantoic fusion.

A mouse model for the study of DNAJB6-related myopathy has recently been established by Bengoechea et al. (2015). These authors generated, under the muscle creatine kinase (MCK) promoter, four different transgenic animals, for both the long and short *DNAJB6a* and *DNAJB6b* isoforms: Two wild-type and two with the most common human mutation F93L. The authors observed that the DNAJB6b-F93L protein levels were higher than those of the wild type protein, and showed that this was due to a slower degradation rate of the mutant protein compared to the wild-type. Bengoechea and coworkers also demonstrated that only the DNAJB6b-F93L mutant construct was able to induce some of the pathological features of the LGMD1D phenotype, such as muscle weakness, myofibrillar disorganization, desmin accumulation, autophagic vacuoles, and aggregation of RNA binding proteins (hnRNAPA1 and hnRNAPA2/B1). Electron microscopy highlighted myofibrillar disorganization, mitochondrial alteration and presence of autophagic vacuoles. Immunostaning showed desmin, keratin 8 and 18 accumulation in muscle fibers, and aggregation of RNA binding proteins (hnRNAPA1 and hnRNAPA2/B1).

Another recent interesting mouse model, by Kakkar et al. (2016) overexpressing the human DNAJB6 protein under control of a brain-specific nestin promoter, although not directly related to muscle diseases, shows the potential of DNAJB6 as therapeutic target in protein aggregate disorders. This mouse was crossed with the R6/2 polyQ mice (HTT), model for Huntington disease. The double transgenic mice HTT/DNAJB6 had a significant reduction of inclusions in the brain only and a better rotarod test performance, when compared to the HTT mice. Since the overexpression of DNAJB6 protein in brain did not show secondary effects in these animals, the authors speculate that DNAJB6 could be a valid therapeutic target in polyQ disorders.

## Clinical features of DNAJB6-related myopathies

**Figure 4:**
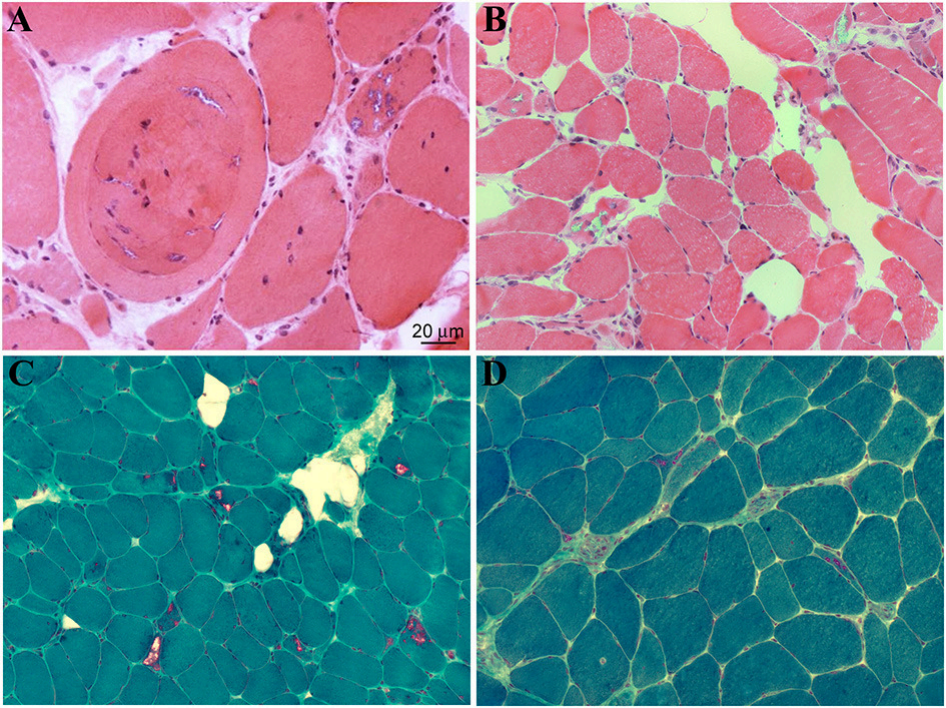
**Histopathological features of muscle biopsies from DNAJB6-mutated patients**. H&E **(A,B)** and Gomori trichrome stains **(C,D)** reveal several autophagic vacuoles, visible either in subsarcolemmal regions or centrally located, internalized myonuclei as well as variability in fiber sizes (Ruggieri et al., 2015).

Clinical features are summarized in Table 1 and histopathological aspects are represented in Figure 4.

**Table 1:** **Clinical and molecular findings in DNAJB6-related families**.

**DNAJB6 mutation**	**References**	**Family ID**	**Ethnicity**	**Age of onset**	**Prevalent weakness distribution**	**EMG**	**Serum CK**	**Muscle pathology**	**Cardiac involvement**	**Loss of ambulation**	**Respiratory failure**	**Bulbar weakness**
c.265T>A p.Phe89Ile	Sarparanta et al., 2012	DUK1047	Caucasian American	20–55	P > D; LL > UL	Myopathic	Normal −10 x	Myopathic, RV, MA	No	na	No	No
c.265T>A p.Phe89Ile	Sarparanta et al., 2012	DUK1701	Caucasian American	14–55	P > D; LL = UL	Myopathic	Normal −3.5 x	Myopathic, not reassessed	No	Yes	No	Yes (3 of 15 patients)
c.265T>A p.Phe89Ile	Suarez-Cedeno et al., 2014	n.d.	Native American/Scottish	<10–adult	P > D; LL = UL; Ax	Myopathic	Normal	RV, intracellular congophilic inclusions	No	Yes (a 50 years old pt with childhood onset)	No	No
c.265T>A p.Phe89Ile	Couthouis et al., 2014	n.d.	Caucasian American	8–18	P > D; LL > UL	na	P1: 181 U/L P2: 3000 U/L	Rimmed vacuoles	No	Yes (the 56 years old proband)	Dyspnea and sleep-disorder breathing	No
c.271T>A p.Phe91Ile	Palmio et al., 2015	Family A	Finnish	6–13	P > D; LL > UL; Ax	Myopathic	Normal −2 x	Myopathic, RV	No	Yes (at age 27 years)	Severe	Yes
c.271T>A p.Phe91Ile	Ruggieri et al., 2015	1s	Italian	16	P > D; LL = UL	Myopathic	Normal	Dystrophic, RV	No	Yes	Mild, non-Progressive	No
c.271T>C p.Phe91Leu	Palmio et al., 2015	B	British	12	P = D; LL > UL; Ax	na	Normal	na, RV	No	Yes	Severe	Yes
c.271T >C p.Phe91Leu	Nam et al., 2015	Family 1	Korean	8–11	P > D; LL > UL	Myopathic	83–245 U/L	Myopathic, RV	Prolonged QT, RBBB, 1st AV block	Yes (a female pt in her 20s)	Mild	Yes
c.273C>G p.Phe91Leu	Ruggieri et al., 2015	2s	Italian	11	P = D; LL = UL	Myopathic	1.2 x	Dystrophic, RV	No	Yes	Nocturnal and daily non-invasive ventilation, cough mechanical assistance	Yes
c.277T>A p.Phe93Ile	Sato et al., 2013	FA	Japanese	30s	P > D; LL > UL	Myopathic	268–1044 U/L	Myopathic, RV	CRBBB in FA-1	No	No	No
c.277T>C p.Phe93Leu	Harms et al., 2012	Family 1	Caucasian	30s	P > D; LL > UL	Myopathic	2–5 x	RV, intracellular congophili cinclusions	No	No (wheelchair use for community mobility 20 years from onset)	No	No
c.277T>C p.Phe93Leu	Sarparanta et al., 2012	IT2	Italian	30–55	P > D; LL > UL	Myopathic	Normal −8 x	Myopathic, RV, MA	No	na	No	No
c.279C>A p.Phe93Leu	Sarparanta et al., 2012	IT1	Italian	20–50	P > D; LL = UL	Myopathic	2 x	Myopathic, RV	No	na	No	No
c.279C>G p.Phe93Leu	Sarparanta et al., 2012	FF1	Finnish	20–60	P > D; LL > UL	Myopathic	Normal −3 x	Myopathic, RV, MA	No	Yes (a 80 years old patient)	No	Yes (1 of 10 pts)
c.279C>G p.Phe93Leu	Sarparanta et al., 2012	FF2	Finnish	45–50	P > D; LL > UL	Myopathic	2–3 x	Myopathic, RV, MA	No	No	No	No
c.279C>G p.Phe93Leu	Sarparanta et al., 2012	FF3	Finnish	38	P > D; LL > UL	Myopathic	3 x	Myopathic, RV, MA	No	No	No	Yes
c.279C>G p.Phe93Leu	Sarparanta et al., 2012	FF4	Finnish	28–40	P > D; LL > UL	Myopathic	Normal −8 x	Myopathic, RV, MA	No	No	No	No
c.279C>G p.Phe93Leu	Sarparanta et al., 2012	FF5	Finnish	40	P > D; LL > UL	Myopathic	Normal	Myopathic, RV, MA	No	No	No	Yes
c.279C>G p.Phe93Leu	Sato et al., 2013	FB	Japanese	25	P > D; LL > UL	na	696 U/L	Myopathic, RV	No	No	No	No
c.279C>G p.Phe93Leu	Sato et al., 2013	FC	Japanese	50s	P > D; LL > UL	na	523 U/L	Myopathic, RV	No	No	No	No
c.279C>G p.Phe93Leu	Sato et al., 2013	FD	Japanese	37–57	P > D; LL > UL	Myopathic	165–251 U/L	Myopathic, RV	No	Yes (a 75 years old patient)	No	No
c.279C>G p. Phe93Leu	Yabe et al., 2014	FD	Japanese	57	P > D; LL > UL	Myopathic	93 U/L	Myopathic, RV	No	Yes	No	No
c.279C>G p.Phe93Leu	Ruggieri et al., 2015	3s	Italian	45	P > D; LL > UL	Myopathic	1.5 x	Myopathic, RV	No	No	No	No
c.287C>G p.Pro96Arg	Harms et al., 2012	Family 2	African American	18–35	D > P; LL = UL	Myopathic	278–339 U/L	na	No	Yes (within 20–30 years from onset)	No	No
c.298T>G p.Phe100Val	Ruggieri et al., 2015	Family 1	Italian	10–50	P = D; LL > UL	Myopathic	1.5–4 x	Dystrophic, RV	No	Yes	Dyspnea	Yes (4 of 5 pts)
c.346+5G>A splicing	Ruggieri et al., 2015	4s	Italian	6	D > P; LL > UL	Myopathic	1.1 x	Dystrophic, RV	No	Yes	Mild	No

*Pt, patient; na, not available; RV, rimmed vacuoles; MA, myofibrillar accumulations; CRBBB, complete right bundle-branch block; RBBB, right bundle branch block; 1st AV block, first degree atrioventricular block; Ax, axial muscles*.

In the first description of the “unusual form of muscular dystrophy” in the paper by Schneiderman et al. (1969), clinical symptoms presented since the third decade of life, manifesting as difficulty in climbing stairs, subsequently followed by proximal upper limb muscles weakness. No bulbar or facial muscle involvement were reported. Disease progression was slow over the years, with only two patients in their seventies being bedridden. Electromyography was normal, as normal were mental abilities. Light microscopy of deltoid muscle biopsies of clinically affected subjects, showed marked variation in fiber size, numerous vacuolated fibers, and slight increase in connective tissue and fat. At the electron microscopy some of the myofibers presented foci of degenerated myofibrils at the center and in the subsarcolemmal regions, which were replaced by granular or vacuoles containing myelin-like bodies, dense lysosomal-like structure, and dilated vesicles.

Subsequent linkage studies on Schneiderman's patients and additional American and Finnish families by several groups (Speer et al., 1995, 1999; Sandell et al., 2010; Hackman et al., 2011) allowed the identification of a common haplotype on 7q36, a region of 3.4 Mb, containing 12 known genes. All the patients were characterized by late onset progressive proximal lower limb muscle weakness with or without proximal upper limb involvement, increased CK levels, absent ankle deep tendon reflexes, and no features suggestive of any other known myopathy. Histology of different lower limb muscles (vastus lateralis, medial gastrocnemius, or soleus) displayed myopathic and dystrophic features, rimmed vacuoles and protein aggregates.

Sequencing of the candidate genes among the 12 present in the linked region, allowed Sarparanta et al. (2012) to identify the genetic cause of LGMD1D in the *DNAJB6* gene. The changes were all point mutations, notably c.279C>G (p.Phe93Leu) in the Finnish families, c.267T>A (p.Phe89Ile) in the American families, and c.279C>A and c.277T>C (p.Phe93Leu) in further Italian families. Interestingly, all the reported mutations were in the exon 5 of the gene, encoding the G/F domain.

The association of these clinical features with mutations in the *DNAJB6* gene and the use of new sequencing technologies allowed the identification of novel mutations in more LGMD1D patients. By exome sequencing in patients that presented with dominant or sporadic myopathy and that were genetically unresolved, Harms et al. (2012) identified *DNAJB6* mutations in two new families, in particular a c.277T>C (p.Phe93Leu) and a c.287C>G (p.Pro96Arg) change. Of the two mutated families though, one Caucasian presented weakness in the limb-girdle muscles, while the second, of African American origin, had predominant lower limb distal weakness, which had never been reported before. The number of published mutations and ethnicity increased with the description of four additional Japanese families by Sato et al. (2013) and two further cases with childhood onset and lower limb proximal muscle weakness described by Suarez-Cedeno et al. (2014) and Couthouis et al. (2014).

Of note, one Japanese patient carrying the Phe93Leu mutation was detailed (Yabe et al., 2014) as having developed fronto-temporal dementia (FTD). His cognitive impairment worsened with age and the patient died at 76 years of age. The immunoreactivity of DNAJB6 in the frontal cortex was greatly reduced as compared with other forms of dementia, but was normal in the thalamus. In the fronto-temporal cortex ubiquitin-positive aggregates were also present. No mutations in any of the known FTD genes were revealed. Based on altered LC3 and p62 immunostaining of the frontal lobe of this patient, the authors suggested a possible impairment of the autophagy pathway such as seen in patients with *VCP* gene mutations. Considering that this suggestion is solely based on histological findings, further investigation to prove its accuracy will be necessary, as stated by the authors themselves.

More recently, the widening of the phenotypic spectrum of *DNAJB6*-related myopathies was confirmed and a tentative genotype-phenotype correlation was hypothesized. In the work by Ruggieri et al. (2015), the authors redefined the genetic locus and some clinical features of a previously reported family with “autosomal dominant vacuolar neuromyopathy” (Servidei et al., 1999). By whole exome sequencing, a novel missense mutation in the *DNAJB6* gene, a c.298T>G (p.Phe100Val), was identified in this family. Again, this mutation affects a conserved amino acid of the G/F domain. Moreover, four sporadic cases were reported, one with a known transition c.279C>G (p.Phe93Leu) and three with novel mutations of the G/F domain: Two missense, c.271T>A (p.Phe91Ile), and c.273C>G (p.Phe91Leu), and one, c.346+5 G>A, leading to an alternative splicing event causing the skipping of exon 5. Clinically the two patients carrying the different mutations of Phe91 had a severe childhood-onset limb-girdle myopathy. The patients with the Phe100Val mutation had distal-onset myopathy, unique early bulbar involvement, and a gender-modified wide age-of-onset range. The patient with the splicing defect that entirely eliminates DNAJB6's G/F domain (ΔG/F), had severe distal childhood-onset myopathy. Muscle imaging revealed that muscles previously considered uninvolved in DNAJB6-myopathy, e.g., lateral gastrocnemii, were affected in patients with new mutations. The authors observed that the mutations affecting the amino acid in the more C-terminal part of the G/F domain (Pro96 and Phe100 in this) as well as the mutation causing skipping of the entire G/F domain were correlating with a more distal phenotype compared to the more N-terminal amino acids (Phe89, Phe91, and Phe93). Furthermore, they highlighted the fact that there seems to be a range of severity correlating with the different *DNAJB6* mutations so far reported, even though more cases are necessary to confirm this hypothesis. In particular, from most severe to least severe LGMD1D phenotype, the mutations are as follow: ΔG/F and Phe91 mutations, Phe100Val, Pro96Arg, and Phe89 mutations and Phe93 mutations.

This genotype-phenotype correlation was once more confirmed by Palmio et al. (2015), reporting a Finnish family and a sporadic British female case with a severe form of LGMD1D with childhood onset, rapidly progressing to loss of walking ability within the end of the third decade, contractures and respiratory failure. The patients carried mutations of the Phe91 amino acid, respectively c.271T>A (p.Phe91Ile) and c.271T>C (p.Phe91Leu). Likewise, the severe phenotype associated to the Phe91Leu was observed by Nam et al. (2015) in a Korean LGMD1D family with childhood onset and fast progression of the symptoms as well as involvement of respiratory and bulbar muscles.

It is intriguing to note that the correlation between genotype and clinical presentation can be seen as well with the use of imaging techniques. In fact, muscle imaging has shown that the pattern of muscle involvement appears to be more variable than previously thought and somewhat different according to the mutated amino acid. Indeed, in 2013 Sandell et al. (2013), published the first analysis of muscle MRI or CT features in 23 Finnish and Italian *DNAJB6* mutated patients. All the patients carried mutations affecting the Phe93 amino acid. The pattern and timing of engagement of the imaged muscles were consistent among all the patients and considered pathognomonic for LGMD1D: At the early stages of the disease the more affected muscles were soleus, adductor magnus, semimembranosus, and biceps femoris, then the medial gastrocnemius and adductor longus, and finally also the vasti muscles of the quadriceps resulted to be compromised. The muscles that were affected only in a very late stage of the disorder were lateral gastrocnemius, rectus femoris, sartorius, gracilis, and the anterolateral muscles of the lower legs. However, it was later on shown that this pattern of involvement was slightly different in patients with the Phe91, Phe100, and ΔG/F changes (Nam et al., 2015; Palmio et al., 2015; Ruggieri et al., 2015). In patients with these mutations, the recti, sartorii, gracilis, and anterolateral muscles were relatively spared, but the lateral gastrocnemii were greatly compromised already in relatively early stages of the disease.

## Conclusions

With the discovery of novel mutations in the *DNAJB6* gene, the spectrum of related phenotypes, in terms of age of onset, severity and group of muscles involved, is expanding. The number and type of mutations (all but one missense) so far appears limited, involving a few amino acids of the G/F domain.

Although our knowledge of the role of DNAJB6 in the pathogenesis of muscle diseases has made great progression, several questions remain unsolved:

1. DNAJB6 is a ubiquitous protein. Therefore, why do *DNAJB6* mutations affect mainly skeletal muscle and rarely the central nervous system (as known so far), and why has no cardiac involvement been reported in these patients? Possible explanations include the selective expression of a DNAJB6 client protein in skeletal muscle, the interaction of DNAJB6 with the CASA system particularly with BAG3 (already known to cause a myofibrillar myopathy), or a functional redundancy of DNAJ family members in all tissues except skeletal muscle.
2. Why are only mutations of the G/F domain implicated? Are any other regions of the protein more flexible and therefore more adaptable to variations, or are mutations in other regions lethal? In this context, the animal models could be useful in preclinical studies addressed to better understand the physio-pathological mechanisms involved in DNAJB6-related myopathies.
3. Is there a function for the DNAJB6a isoform in muscle? It is known that it reduces malignancy in several forms of cancer by acting on AKT and β catenin pathways (Yu et al., 2015). Could this be related to a possible role of DNAJB6a in fine-tuning of critical cellular signaling also in muscle?

The clarification of these issues and of the molecular mechanisms driven by DNAJB6 protein will provide the knowledge for implementing therapeutic strategies for patients with DNAJB6-related myopathies, as well as for patients with neurodegenerative diseases caused by toxic protein aggregation.

## Author contributions

MM and AR conceived the review focus, conducted literature review, summarized, and finalized the manuscript. AR, SS, and SZ reviewed literature, wrote first draft, and finalized the manuscript. BP and LM revised and finalized the clinical part of the manuscript. All authors approved final version of manuscript.

## Funding

This work was supported by Institutional funds from the Italian Ministry of Health.

### Conflict of interest statement

The authors declare that the research was conducted in the absence of any commercial or financial relationships that could be construed as a potential conflict of interest.
